# Recent advances in understanding and treating ARDS

**DOI:** 10.12688/f1000research.7646.1

**Published:** 2016-04-22

**Authors:** Rebecca M. Baron, Bruce D. Levy

**Affiliations:** 1 Division of Pulmonary and Critical Care Medicine, Brigham and Women’s Hospital, Harvard Medical School, Boston, Massachusetts, USA

**Keywords:** Adjunctive therapy, Berlin criteria, Low Tidal Volume Ventilation, Prone Positioning, Neuromuscular Blockade

## Abstract

Acute respiratory distress syndrome represents a complex syndrome with considerable morbidity and mortality, for which there exist no targeted treatment strategies. However, recent advances in clinical care have improved outcomes, and we will review a number of these approaches here, as well as explore the mechanisms underlying the benefit of intervention that might point us in the direction toward future treatment and preventive strategies for this devastating syndrome.

## Introduction

The acute respiratory distress syndrome (ARDS), first described in 1967, remains difficult to treat and has significant morbidity and mortality. Risk factors for ARDS include factors resulting from direct injury to the lung (e.g., pneumonia and gastric aspiration) or indirect injury (e.g., sepsis and pancreatitis). These conditions result in inflammatory lung injury and hypoxemia that arise from disruption of the alveolar-capillary membrane and influx of protein-rich edema fluid, producing physiologic lung dysfunction. Remarkably, despite intense investigation and numerous large-scale clinical trials, no targeted medical therapies have yet been developed nor proven effective, and there exist no universally agreed-upon biomarkers that might predict severity of illness, or clinical outcomes, or both. These challenges in characterization and treatment likely result from the heterogeneity of ARDS as well as the difficulty of treating a “syndrome” rather than a molecularly confirmed disease; however, a number of management strategies have proven beneficial and have resulted in reductions in mortality ^1^. It is unlikely, as is the case for many serious ailments, that there is a “one size fits all” treatment for ARDS, and thus improved understanding of the disease process and appropriately characterizing severity of illness will be critical for making advances in treatment strategy. Furthermore, the National Institutes of Health (NIH) has recognized the importance of addressing strategies to prevent ARDS development, thus forming the new version of the ARDS Network under the heading of “PETAL”: Prevention and Early Treatment of Acute Lung Injury. Therefore, in this brief review, we set out to discuss some of the recent advances in understanding and treating ARDS, as well as to address the possible biological mechanisms underlying these mechanisms, to attempt to shed light on potential areas for future scientific investigation. We focus our discussion on significant articles that have modified mortality from ARDS, and we summarize a proposed overall approach to ARDS.

## Advances in defining acute respiratory distress syndrome and improving mortality prediction: the Berlin Criteria

From 1994 to 2012, ARDS was defined on the basis of the American-European Consensus Conference (AECC) criteria: (a) acute onset of hypoxemia defined by partial pressure of arterial oxygen/fraction of inspired oxygen (P _a_O _2_/F _i_O _2_, or P/F) ratio of >200 with (b) new bilateral infiltrates (c) not attributable to heart failure as defined by pulmonary capillary wedge pressure (PCWP) (as measured by a Swan-Ganz catheter) of not more than 18 mmHg (or absence of suspected left atrial hypertension/cardiogenic pulmonary edema if PCWP was not available) ^2^. These criteria were adopted with the goal of more uniformly defining the syndrome and identifying appropriate patients for ARDS therapies and enrollment in clinical trials. Although these criteria facilitated these goals, it was felt that certain improvements in the definition might improve the clinical phenotyping and risk stratification of ARDS subjects, including a more explicit definition of “acute” onset of hypoxemia, further definition of the dependence of the P/F ratio on ventilator settings (in particular, the positive end-expiratory pressure (PEEP) setting), improved criteria for chest radiograph interpretation of “bilateral infiltrates”, and more explicit guidance with defining the contribution of cardiogenic pulmonary edema to the clinical picture.

In 2012, the European Society of Intensive Care Medicine convened an expert panel to improve the reliability and validity of the ARDS definition, termed the Berlin Criteria ^3^. These newer criteria improve upon the old, in part through the following: (a) defining three categories of ARDS severity on the basis of P/F ratio: P/F ratio ≤300 and >200 (“mild” ARDS, which was previously categorized as acute lung injury under AECC criteria), P/F ratio of between 100 and 200 (“moderate” ARDS), and P/F ratio of <100 (“severe” ARDS); (b) defining “acute” onset of bilateral infiltrates as within 7 days of exposure to an ARDS risk factor or worsening respiratory symptoms; (c) more definitive chest radiograph criteria were provided (with retention of the description of bilateral infiltrates consistent with pulmonary edema and not fully explained by effusions, lobar/lung collapse, or nodules), and use of chest computed tomography was allowed for fulfilling the radiographic definition as well; (d) use of the PCWP for defining cardiogenic pulmonary edema was removed (given the declining use of Swan-Ganz catheters), and it was acknowledged that cardiogenic and non-cardiogenic pulmonary edema can coexist. However, determination of whether the bilateral infiltrates are attributable to a cardiogenic cause cannot be based solely upon clinical decision making. If a risk factor for ARDS is not identified, then some objective criteria of cardiac function, such as echocardiography, are required to exclude a sole cardiogenic cause for pulmonary edema; and (e) minimum use of PEEP of at least 5 cm H _2_O on the mechanical ventilator (or delivered by non-invasive ventilation only in the mild ARDS category) in assessing the severity of oxygenation impairment using the P/F ratio.

### Implications of the Berlin Criteria

The Berlin Criteria were derived and validated, and additional variables that were considered in the definition (lung compliance, radiographic severity, levels of PEEP, and exhaled minute ventilation) did not improve severity prediction and therefore were not incorporated into the final criteria (although the authors acknowledged that analysis of these factors plays an important role in bedside clinical care). Of note, defining the “severe ARDS” category called attention to the most afflicted group (with a P/F ratio of <100), which has the highest mortality irrespective of ventilator strategy and therefore might benefit from applications of more advanced ARDS rescue strategies (see discussions below). Specifically, mortality rates in the mild, moderate, and severe groups were 27%, 32%, and 45%, respectively, and the Berlin Criteria improved mortality prediction beyond that of AECC; however, as acknowledged by the authors, there are clearly limitations to clinical criteria in defining a syndrome ^1, 3, 4^. Ideally, the use of additional biologic predictors might have the capacity to improve prediction of outcome and risk stratification. Although the hunt for predictive biomarkers for ARDS development and outcome is still under way, existing studies suggest the promise of using plasma biomarkers—e.g., interleukin-8 (IL-8), tumor necrosis factor alpha (TNFα), surfactant protein-D (SPD), and mitochondrial DNA—to improve prediction of outcomes beyond clinical classification algorithms (e.g., Acute Physiology and Chronic Health Evaluation scoring systems) ^5, 6^. Recent studies suggest that ARDS might be better predicted by specific biomarkers, such as plasma levels of the soluble form of the receptor for advanced glycation end products (sRAGE) as a marker of lung epithelial injury ^7, 8^ and plasma levels of tumor necrosis factor receptor-1 (TNFR1), IL-6, IL-8, and plasminogen activator inhibitor-1 (PAI1) as markers of a hyperinflammatory ARDS subphenotype ^8, 9^. Although the Berlin Criteria have enhanced our clinical phenotyping systems, ongoing work in clinical/biological phenotyping of critically ill subjects will ideally facilitate additional prevention trials, allowing investigators to target specific risk groups with modifiable risk factors for ARDS development.

## Advances in mechanical ventilation support of patients with acute respiratory distress syndrome: low tidal volume ventilation

In a seminal study performed by the ARDS Network in 2000, mechanical ventilatory support of ARDS patients with 12 ml/kg (ideal body weight) tidal volume was compared with low tidal volume ventilation at 6 ml/kg, and there was a significant reduction in mortality with low tidal volume ventilation (38% to 31%) ^10^. This study prompted the widespread use of low tidal volume ventilation in supporting patients with ARDS and has led to ongoing studies to investigate the mechanisms underlying this profound benefit (see below). In addition, this important trial has prompted additional recent trials investigating whether low tidal volume ventilation might also benefit other populations of patients, such as those undergoing mechanical ventilation for an operative procedure (i.e. whether patients without significant pre-existing lung injury might be similarly injured with potentially injurious mechanical ventilator settings, thereby being placed at risk for the development of ARDS).This question remains a point of clinical debate in setting mechanical ventilation parameters for critically ill patients without the presence of ARDS. Although it is known that normal laboratory mice exposed to high tidal volume ventilation develop lung injury ^11^, it is not clear whether this is the case for humans without pre-existing lung injury. Interestingly, a recent article reported that the use of a “prophylactic” protective ventilation strategy improved clinical outcomes (relative to higher tidal volumes usually used during anesthesia in patients without pre-existing lung injury with the goal of preventing atelectasis) in intermediate- and high-risk patients undergoing abdominal surgery ^12^.

### Potential mechanisms underlying the protective nature of low tidal volume ventilation

The findings of improved mortality with low tidal volume ventilation prompted widespread investigation into the mechanisms underlying this protection, resulting in a vast expansion in our understanding of factors driving mechanotransduction-related lung injury. Physiologic lung improvements as a result of low tidal volume ventilation have been attributed to a number of factors, including most grossly, reduced incidence of barotrauma (application of high pressures to the lung resulting in injury), volutrauma (application of high tidal volumes—i.e. lung stretching—resulting in injury), and perhaps improved hemodynamics (blood pressure and organ perfusion) as a result of less overdistention of the lung and improved venous return to the heart; however, ventilation at low tidal volumes can result in collapse of the lung parenchyma, and trials of high-frequency oscillatory ventilation that allowed for very small tidal volumes did not prove beneficial ^13, 14^, supporting the complexity of ARDS pathophysiology and management. Mechanical ventilation without maintenance of open lung units has the potential to exacerbate lung injury as a result of opening and closing of lung units, termed “atelectrauma” ^15, 16^, which has led to widespread studies of optimal application of PEEP to maintain open lung units, and a recent meta-analysis suggested that higher levels of applied PEEP might be beneficial in patients with moderate ARDS ^17^. Beyond physiologic benefits of low tidal volume ventilation, numerous studies have called attention to the concept of “biotrauma” as a result of injurious mechanical ventilation, in which stretching of lung units might activate cellular signaling cascades resulting in lung inflammation, increased release of pro-inflammatory mediators (e.g., IL-6), and effects on non-pulmonary tissues resulting in multi-system organ failure. Potential physiologic benefits of low tidal volume ventilation have recently been reviewed in detail ^19^.

## Advances in adjunctive therapy for severe acute respiratory distress syndrome: neuromuscular blockade

Neuromuscular blockers (NMBs) have been used for a long period of time in the intensive care unit, largely to facilitate mechanical ventilation of ARDS subjects when sedation alone was insufficient, usually in the setting of severe gas exchange impairment, or to facilitate other advanced therapies for ARDS, such as prone positioning (see below), or to do both; however, protocolized care for use of NMBs in ARDS has not been uniformly applied, and concerns regarding adverse effects of NMBs (e.g., prolonged neuromuscular weakness) without clear data showing benefit of NMBs limited widespread use until recently. Papazian *et al.* examined 340 intubated patients in a multi-center trial with severe ARDS (P/F ratio of <150), who were randomly assigned to receive NMB (cisatracurium besylate) versus placebo for 48 hours. All patients received low tidal volume ventilation and were on at least 5 cm H _2_O PEEP. The adjusted 90-day in-hospital mortality rate was lower with NMB versus placebo, and no increased neuromuscular weakness was observed in the NMB group ^20^. Additionally, an increased number of ventilator-free days was observed in the NMB group. Of note, both groups received deep sedation. This study has raised important questions about the utility of NMB in ARDS, and there is sufficient uncertainty about its widespread use that the NIH PETAL Network is addressing this issue in one of its first network trials. Thus, further data will be available in the future to help guide clinical practice. Residual questions that remain include whether patients solely with severe ARDS might benefit from NMB, what the optimal NMB infusion duration might be, whether similar benefits might be observed with NMB agents other than cisatracurium besylate, and what the independent effects of heavy sedation apart from NMB might be ^21, 22^.

### Potential mechanisms underlying the benefit of neuromuscular blockade

Although much of the mechanism remains to be learned regarding protective effects underlying NMB (and additional significant information is likely to be gained from the upcoming PETAL Network NMB trial and planned associated ancillary studies), there exist data to support a number of possible pathways of benefit: (a) NMBs counteract patients bucking the ventilator, thereby limiting lung injury arising from ventilator dyssynchrony—of note, an increased rate of pneumothoraces (consistent with barotrauma) was observed in the placebo group ^20^; (b) NMBs might result in less biotrauma as evidenced by less end-organ failure associated with their use ^20^, as well as reduction in lung (IL-1β, IL-6, and IL-8) and serum (IL-1β and IL-8) cytokines in patients on NMB ^23^; (c) NMBs limit expiratory muscle function and therefore reduced respiratory system collapse and derecruitment that might result in improved respiratory system compliance and improved ventilation-perfusion matching ^21, 22^. Interestingly, a recent preclinical study suggests that the mechanism of protection of NMBs might relate to direct anti-inflammatory effects of blocking the nicotinic acetylcholine receptor-α1, independent of effects on improving ventilator dyssynchrony ^24^.

## Advances in adjunctive therapy for severe acute respiratory distress syndrome: prone positioning

Although it was realized in the 1970s that prone positioning improved oxygenation in ARDS, numerous studies over ensuing decades demonstrated improvement in oxygenation but failed to show improved mortality from prone positioning. This lack of mortality benefit, coupled with concerns regarding possible adverse events from proning patients (e.g., facial edema, skin breakdown at areas of pressure necrosis, transient desaturation as well as less commonly dislodgement of lines, endotracheal tubes, and hemodynamic instability), led to limited/sporadic use across clinical ARDS centers ^25^. In 2013, Guérin *et al.* examined prone positioning (at least 16 hours per day) versus standard positioning in 466 subjects with severe ARDS (within 36 hours of intubation and after a stabilization period of 12 to 24 hours; P/F ratio of < 150), F _i_O _2_ of at least 0.6, low tidal volume ventilation, and PEEP of at least 5 cm H _2_O and found a striking 28-day mortality benefit of prone positioning (32.8% supine versus 16% prone), and a mortality benefit persisted until day 90 ^26^. Of note, there was no significant difference in complications between the prone and supine groups (except for an increased rate of cardiac arrests in the supine group); however, the authors acknowledge that this study was carried out in centers with substantial experience and expertise in prone positioning. This lack of expertise in prone positioning, the potential complications that might arise from proning, and the potential difficulty in selecting optimal patients who might benefit from proning (a highly selected group of patients was included in the most recent trial) have led to variability in uniform adoption of prone positioning in ARDS clinical centers.

### Potential mechanisms underlying the benefit of prone positioning

Although a number of studies over many years examined potential benefits of proning, a mortality benefit to the degree described above was only recently observed. Some of the earlier studies were small studies and in addition studied patients in later ARDS phases when prone positioning might be less likely to reverse the disease process. It is possible that more routine use of low tidal volume ventilation in this most recent study is a factor, as well as the more prolonged period of proning that was employed in this study compared with other trials ^27^. Of note, differential use of neuromuscular blockade (increased NMB was used in the prone position group) has been cited as an important factor to consider in interpreting this trial (see **Advances in adjunctive therapy for severe acute respiratory distress syndrome: neuromuscular blockade** section above). Some earlier studies suggested that the most afflicted patients (i.e. those with the lowest P/F ratios) might benefit from proning, prompting the selection of the patient population in the most recent study ^26^. In general, potential benefits of proning might include (a) improved lung ventilation perfusion matching, (b) improved right ventricular dysfunction ^28, 29^, and (c) recruitment of lower-lobe atelectatic lung units (perhaps related to reduced compression of lung units in the prone position) and decreased intrapulmonary shunting, as well as potentially improved maintenance of open lung units, thus limiting ventilator-induced lung injury (through mitigating repeated opening and closing of lung units that generates lung injury). It is believed that some patients also experience improved secretion clearance gravitationally in the prone position ^25^.

## Conclusions

Although ARDS represents a complex syndrome with considerable morbidity and mortality, recent advances in clinical care have improved outcomes, as described in this review. Support of ARDS patients with low tidal volume ventilation has become the standard of care, and this approach has revealed important underlying mechanisms that have led to new areas of investigation in lung injury. Use of the Berlin Criteria has aided in the identification of the most afflicted ARDS patients who might benefit from rescue therapies, and targeting of neuromuscular blockade and prone positioning in severe ARDS has recently proven beneficial in terms of improved ARDS mortality. Ongoing studies will be important for providing additional information for helping us target these modalities to the patients most likely to benefit from them as well as to gain further understanding of the mechanisms underlying benefit of these modalities. An additional area of clinical care and investigation not reviewed here in detail is the use of conservative fluid strategy to decrease ventilator time in patients with ARDS ^30, 31^, although more recent data have called attention to the possibility that a more restrictive fluid management strategy is associated with cognitive dysfunction ^32^. Fluid management in critical illness is currently under review ^33^, and the information that is gleaned may help guide clinical practice in the future.

Although targeted medical therapies have not yet proven beneficial in clinical trials, promising targets ^16, 34^, including those in an ongoing NIH-funded trial in mesenchymal stromal cells ^35, 36^, are under investigation; however, it is widely appreciated that the heterogeneity of the syndrome might require a more targeted/personalized approach toward ameliorating complex biologic pathways that might be differentially activated with different host responses and at different time points in each illness. Increasingly, it is appreciated that efforts targeted at prevention of ARDS represent a growing opportunity for investigation and treatment ^37^, both in optimally identifying at-risk subjects and in selecting those most likely to benefit from early interventions. Of note, prevention studies will be a major focus of the NIH PETAL Network. In conclusion, although ARDS represents a challenging syndrome to characterize, manage, and treat, recent advances have improved clinical outcomes, and exciting approaches on the horizon hold promise for allowing us to gain insights into novel treatment strategies.
